# Eric Co. Med. Society

**Published:** 1847-09

**Authors:** 


*Erie Co. Med. Society.*—We omitted in a previous notice of the meeting of the Erie Co. Med. Society held in June last, to state that a committee was appointed to revise the Constitution and By-laws of the Society. The attendance at the stated meetings of the society has hitherto been small, especially of members from the country. Several whose presence would be very acceptable, and add much to the character and usefulness of the meetings, have been absent for years. It is understood that objections exist, in the minds of some, to the requirements of the Constitution and By-laws. We trust the committee who have in charge their revision, will remedy what is amiss, and remove all obstacles in the way of an universal and cordial interest in the prosperity of the society.

